# Women’s 2023 and 2024 WXV international rugby competitions: injury surveillance study

**DOI:** 10.3389/fspor.2026.1745865

**Published:** 2026-01-29

**Authors:** Colin W. Fuller, Aileen Taylor

**Affiliations:** 1 Colin Fuller Consultancy Ltd, Sutton Bonington, United Kingdom; 2 Colin Fuller Consultancy Ltd, Nairobi, Kenya

**Keywords:** burden, injury, nature, prevention, risk

## Abstract

**Purpose:**

The popularity of women's international rugby has grown significantly over the past decade. The impact of this growth on player welfare, however, has not yet been assessed. The aim of this study was to increase the level of epidemiological information available for women's international rugby-15s.

**Methods:**

Anthropometric and injury data were reported by the medical staff working with 20 national teams taking part in the 2023 and 2024 WXV international rugby competitions. Data related to the incidence, severity, burden, nature and cause of injuries were collected according to the international consensus statement for conducting injury surveillance studies in rugby.

**Results:**

The overall incidence of injury was 49.5 injuries/1,000 player-match-hours with a mean severity of 42.2 days. As two-thirds of all injuries were sustained during the second half of matches, player fatigue is a probable injury risk factor in women's rugby. Concussion (23.3%) and knee-ligament (16.3%) injuries were the most common injuries sustained by backs; whereas, knee-ligament (15.5%) and ankle-ligament (14.0%) injuries were the most common for forwards. Knee-ligament injuries were responsible for most time-loss by both backs (36.8%) and forwards (60.2%).

**Discussion:**

The risk of injury in rugby is defined by the incidence and nature of injuries sustained by players together with the match and training load that players experience. Results obtained from this study enhance the evidence-base available for identifying injury risk factors and developing mitigation priorities for women's rugby-15s.

## Introduction

World Rugby's 2025 report (1) entitled ‘A Blueprint for Growth: Women's Rugby Fan, Data and Commercial Insights’ highlighted the worldwide growth and professionalisation of women's rugby over the last decade. To ensure that female-specific issues in rugby were effectively identified and addressed during this period of growth, World Rugby established a Women's Player Welfare Working Group in 2021 (2). This Working Group created a Women's Health section within World Rugby's Player Welfare web pages to provide information relating specifically to women's players' health. A key objective identified by the Working Group was “*to use injury surveillance studies and game analysis reports to track the evolution of the women's game*” (2). World Rugby has long recognised the benefits of implementing robust injury surveillance studies (ISS) (3, 4) to quantify the risks of injury in rugby and to create an evidence-base from which to develop player-welfare programmes. Results from ISS implemented in men's international rugby-7s (5–8) and rugby-15s competitions (9–15) have been published on a regular basis over the past 20 years. Published epidemiological information about women's international rugby, however, has focussed on rugby-7s competitions (7, 16) with information about international rugby-15s restricted to the 2006 (17) and 2010 (18) women's Rugby World Cup (WRWC). Establishing a greater depth of epidemiological data is, therefore, essential for women's rugby in order to support World Rugby's objectives of providing a female-focussed player welfare information hub and tracking changes in the incidence, severity and nature of injuries sustained in women's international rugby.

In 2023, World Rugby established a 3-tier annual WXV rugby-15s competition consisting of three performance levels (WXV1 > WXV2 > WXV3) (19). The purpose of this new competition was to increase competition opportunities for women's national rugby teams and to provide qualifying pathways for the expanded 2025 WRWC. To support the Women's Player Welfare Working Group's aim of using injury surveillance to monitor the evolution of the women's game, World Rugby implemented an ISS covering the 2023 and 2024 WXV competitions. The primary objective of this ISS was to augment the limited epidemiological information available about the incidence, severity, nature and causes of match injuries sustained in women's international rugby. Secondary objectives of the study were to determine whether injury risks have changed, as women's international rugby has developed and become more professional, and to assess how injury risks in women's international rugby-15s compare with men's international rugby.

## Materials and methods

### Participants

Six international teams competed in cross-pool formats in each tier of the WXV competition. Over the 2023 and 2024 study period, this competition structure led to 108 WXV team-games being played. On completion of the 2023 competition, promotions and relegations of some teams took place between the three tiers and, in addition, the three bottom teams in the WXV3 tier were replaced by three new teams for the 2024 WXV competition. In total, twenty-one national teams were involved in the two WXV competitions; twenty of these teams provided anthropometric and injury data, which were analysed and included in this study. The competitions were played in New Zealand (WXV1), South Africa (WXV2) and Dubai (WXV3) in 2023 and in Canada (WXV1), South Africa (WXV2) and Dubai (WXV3) in 2024. All aspects of the ISS were conducted in accordance with the definitions and protocols described in the World Rugby approved consensus statement for ISS in rugby union (3). Anthropometric and injury data were recorded and reported prospectively by each team's medical staff. The studies were approved by World Rugby's Institutional Ethics Committee, as part of their ongoing player welfare management programme. Players approved the collection, analysis and reporting of their injury data in a generalised, unidentifiable format. The present study relates to time-loss match injuries in women's international rugby. The study does not report time-loss injuries sustained during training activities, non-time-loss injuries or health issues related to women's rugby.

### Data collection

All players' anthropometric and injury data were recorded using World Rugby's secure ISS web app. All submitted data were reviewed by the authors for completeness and, if required, followed up with team medical staff prior to analysis.

Players' anthropometric profiles (playing position: back, forward; date of birth; body mass: Kg; stature: cm) were recorded prior to the start of each competition. Injuries were defined as ‘*an injury sustained during a WXV match that prevented a player from taking a full part in all normal training activities and/or match play for more than one day following the day of injury’*. Injuries were classified by location, type, cause and activity associated with injury (3); an appropriate OSICS Code (20) was assigned to each injury. The period in the game when players were injured (0–20, 21–40+, 41–60, 61–80 + minutes) and whether injured players were removed from the game when injured (immediately, later-in-the-game, not-at-all) were also recorded. Injury severity was defined by the number of days a player was injured; players were deemed to be ‘injured’ until they could undertake normal training and be available for match selection. Return-to-play dates for players with injuries unresolved after 90 days were based on the injured player's medical staff's knowledge of the injury, the injury's treatment and rehabilitation and the medical staff's clinical judgement. Injury burden was calculated as the product of injury incidence and mean severity (21–24). Match exposures were based on 15 players (backs: 7; forwards: 8) being exposed for 80 min per team-game.

### Statistical analysis

Unpaired *t*-tests were used to compare players' anthropometric data. Incidence, mean severity and proportions of injuries were compared using *z*-tests; median severity values were compared using the Mann–Whitney *U*-test (25). Distributions of injury locations and types and match activities associated with injuries sustained by backs and forwards were compared using 95% confidence intervals (25, 26). Statistical significance associated with a test was accepted at the *p* ≤ 0.05 level. It is accepted that this approach can conclude that some differences are statistically significant when they actually occurred by chance; for this reason, exact *p*-values are reported for statistical tests. If the specific data required for cross-study comparisons were not included in the study's published research paper, the information required was retrieved from the authors' original ISS injury database.

## Results

### Participant characteristics

Five hundred and eleven players were included from the 2023 WXV competition (backs: 227, forwards: 284) and 517 players from the 2024 WXV competition (backs: 227, forwards: 290). Table 1 summarises the average age, stature and body mass of these players; forwards were significantly taller (*p* < 0.001), heavier (*p* < 0.001) and older (*p* < 0.001) than the backs. Overall, WXV1 players (mean: 170.7 cm) were taller (*p* < 0.001) than WXV2 players (mean: 168.4 cm), who were taller (*p* = 0.447) than WXV3 players (mean: 168.0 cm). WXV1 players (mean; 78.6 Kg) were also heavier (*p* < 0.061) than WXV2 players (mean: 76.9 Kg), who were heavier (*p* = 0.070) than WXV3 players (mean: 75.0 Kg). There were no significant differences in the average anthropometric characteristics of injured players (stature: mean 169.5 cm, *p* = 0.575, body mass: mean 77.0 Kg, *p* = 1.000; age: mean 25.4 years, *p* = 1.000) compared to the values for the overall sample population.

**Table 1:** Anthropometric data for backs and forwards.

Measure	Mean (number of players, standard deviation)		
	Backs	Forwards	ALL players
Stature, cm	166.5 (450, 5.9)	171.1 (565, 6.8)	169.1 (1015, 6.8)
Body mass, Kg	68.4 (449, 8.0)	83.9 (556, 11.9)	77.0 (1005, 12.9)
Age, years	24.9 (454, 4.1)	25.8 (574, 4.3)	25.4 (1028, 4.2)

### Match injuries

The total numbers of injuries sustained by backs, forwards and all players over the two competitions are summarised in Table 2. Fifty-nine of the 101 injuries reported were sustained during the 2023 WXV competition (backs: 22; forwards: 37) and 42 during the 2024 WXV competition (backs: 21; forwards: 21). Five of the 101 injuries reported were recorded as recurrences (backs: 2; forwards: 3). Two-thirds of the injuries sustained occurred in the second half of matches for both backs (first half: 32.6%; second half: 67.4%; *p* < 0.001) and forwards (first half: 33.9%; second half: 66.1%; *p* < 0.001). Thirty-seven per cent of all injured players were removed from play immediately, 23.0% were removed later in the game and 40% were not removed and completed the game. For concussion injuries, 66.7% of injured players were removed from play immediately, 20.0% were removed later in the game and 13.3% completed the game. Players sustaining a concussion were significantly (*p* = 0.009) more likely to be removed from play immediately than players sustaining any other injury.

**Table 2:** Number, exposure (player-hours), incidence (injuries/1000 player-match-hours, 95% CI), mean and median severity (days, 95% CI) and injury burden (days-absence/1000 player-match-hours, 95% CI) of match injuries sustained by backs and forwards.

Measure	Backs	Forwards	ALL players
Injuries	43	58	101
Exposure	952	1088	2040
Incidence	45.2 (33.5–60.9)	53.3 (41.2–69.0)	49.5 (40.7–60.2)
Severity, mean	35.2 (16.1–54.2)	47.4 (27.5–67.3)	42.2 (28.2–56.2)
Severity, median	15.0 (9.0–26.0)	16.5 (7.0–30.0)	16.0 (11.0–21.0)
Injury burden	1589 (1179–2143)	2527 (1953–3268)	2089 (1719–2539)

Table 2 also shows match exposures, mean and median injury severities, injury incidences and injury burdens for backs, forwards and all players. The 101 injuries sustained led to a total of 4,262 player-days-absence (backs: 1,513; forwards: 2,749). The incidence of injury for forwards was higher than that for backs but the difference was not statistically significant (*p* = 0.412). The mean and median severity values for forwards were both higher than those recorded for backs but the differences were again not statistically significant (mean: *p* = 0.385; median: *p* = 0.926). Of the 10 major (>90 days severity) injuries sustained (backs: 2; forwards: 8), six (backs: 1; forwards: 5) were anterior or posterior cruciate ligament injuries. The injury incidence and mean severity values for forwards combined to give a statistically significant (*p* = 0.021) higher injury burden for forwards compared to backs.

The locations and types of injuries sustained during the two WXV competitions are summarised in Table 3. Based on the 95% confidence intervals, there are no statistically significant differences between backs and forwards for either the sub-locations or the sub-types of injury sustained. For backs, concussion (23.3%), knee-ligament (16.3%), ankle-ligament (7.0%) and lower leg-muscle rupture (7.0%) injuries were responsible for 53.5% of injuries. For forwards, knee-ligament (15.5%), ankle-ligament (10.3%), concussion (8.6%) and lower leg-muscle rupture (6.9%) injuries were responsible for 41.4% of injuries. However, knee-ligament injuries were responsible for 36.8%, concussion for 12.6%, knee-meniscus injuries for 11.2% and foot fractures for 5.1% of time-loss for backs. For forwards, knee-ligament injuries were responsible for 60.2% of time-loss, ankle-ligament injuries for 5.6%, foot-fractures for 3.4% and shoulder-dislocations for 3.4%. No catastrophic, career-ending or female-specific injury/medical conditions were reported.

**Table 3:** Locations and types of match injury sustained by backs and forwards.

Injury location/type	% (95% confidence interval)		
	Backs	Forwards	ALL players
INJURY LOCATION -			
Head/neck	34.9 (20.6–49.1)	17.2 (7.5–27.0)	24.8 (16.3–33.2)
Head/face	32.6 (18.6–46.6)	15.5 (6.2–24.8)	22.8 (14.6–31.0)
Neck/cerv^l^ spine	2.3 (0–6.8)	1.7 (0–5.1)	2.0 (0–4.7)
Upper limbs	11.6 (2.0–21.2)	22.4 (11.7–33.1)	17.8 (10.4–25.3)
Shoulder/clavicle	4.7 (0–10.9)	12.1 (3.7–20.5)	8.9 (3.4–14.5)
Upper arm	0.0 (-)	0.0 (-)	0.0 (-)
Elbow	0.0 (-)	3.4 (0–8.1)	2.0 (0–4.7)
Forearm	0.0 (-)	0.0 (-)	0.0 (-)
Wrist/hand	7.0 (0–14.6)	6.9 (0.4–13.4)	6.9 (2.0–11.9)
Trunk	4.7 (0–10.9)	1.7 (0–5.1)	3.0 (0–6.3)
Ribs/upper back	2.3 (0–6.8)	1.7 (0–5.1)	2.0 (0–4.7)
Abdomen	0.0 (-)	0.0 (-)	0.0 (-)
Lower back	2.3 (0–6.8)	0.0 (-)	1.0 (0–2.9)
Sacrum/pelvis	0.0 (-)	0.0 (-)	0.0 (-)
Lower limbs	48.8 (33.9–63.6)	58.6 (45.9–71.3)	54.5 (44.7–64.2)
Hip/groin	4.7 (0–10.9)	1.7 (0–5.1)	3.0 (0–6.3)
Thigh, anterior	0.0 (-)	1.7 (0–5.1)	1.0 (0–2.9)
Thigh, posterior	0.0 (-)	1.7 (0–5.1)	1.0 (0–2.9)
Knee	20.9 (8.8–33.1)	24.1 (13.1–35.2)	22.8 (14.6–31.0)
L-leg/Achilles	7.0 (0–14.6)	6.9 (0.4–13.4)	6.9 (2.0–11.9)
Ankle	9.3 (0.6–18.0)	15.5 (6.2–24.8)	12.9 (6.3–19.4)
Foot/toe	7.0 (0–14.6)	6.9 (0.4–13.4)	6.9 (2.0–11.9)
Injury type -			
Bone	4.7 (0–10.9)	6.9 (0.4–13.4)	5.9 (1.3–10.6)
Fracture	4.7 (0–10.9)	5.2 (0 -10.9)	5.0 (0.7–9.2)
Other bone	0.0 (-)	1.7 (0–5.1)	1.0 (0–2.9)
C/PNS	25.6 (12.5–38.6)	8.6 (1.4–15.8)	15.8 (8.7–23.0)
Concussion	23.3 (10.6–35.9)	8.6 (1.4–15.8)	14.9 (7.9–21.8)
Nerve	2.3 (0–6.8)	0.0 (-)	1.0 (0–2.9)
Joint (non-bone)/lig^t^	41.9 (27.1–56.6)	55.2 (42.4–68.0)	49.5 (39.8–59.3)
Dislocation/sublux^n^	7.0 (0–14.6)	10.3 (2.5–18.2)	8.9 (3.4–14.5)
Lesion meniscus	2.3 (0–6.8)	6.9 (0.4–13.4)	5.0 (0.7–9.2)
Sprain/ligament	32.6 (18.6–46.6)	37.9 (25.4–50.4)	35.6 (26.3–45.0)
Muscle/tendon	23.3 (10.6–35.9)	19.0 (8.9–29.1)	20.8 (12.9–28.7)
Haematoma/etc	11.6 (2.0–21.2)	5.2 (0 -10.9)	7.9 (2.7–13.2)
Muscle rupture/etc	11.6 (2.0–21.2)	8.6 (1.4–15.8)	9.9 (4.1–15.7)
Tendon injury/etc	0.0 (-)	5.2 (0 -10.9)	3.0 (0–6.3)
Skin	2.3 (0–6.8)	5.2 (0 -10.9)	4.0 (0.2–7.8)
Abrasion	0.0 (-)	0.0 (-)	0.0 (-)
Laceration	2.3 (0–6.8)	5.2 (0 -10.9)	4.0 (0.2–7.8)
Other injuries	2.3 (0–6.8)	5.2 (0 -10.9)	4.0 (0.2–7.8)

C/PNS, Central and peripheral nervous system.

### Risk factors

There was no significant difference (*p* = 0.617) between the nature of injuries sustained by backs (acute: 95.0%, 95% CI: 88.2%–100%; gradual onset: 5.0%, 95% CI: 0–11.8%) and forwards (acute: 93.0%, 95% CI: 86.4–99.6%; gradual onset: 7.0%, 95% CI: 0.4–13.6%). The majority of injuries (87.1%) resulted from contact match activities; there was no significant difference (*p* = 0.803) between backs (contact: 92.1%, 95% CI: 83.5%–100%; non-contact: 7.9%, 95% CI: 0–16.5%) and forwards (contact: 83.6%, 95% CI: 73.9–93.4%; non-contact: 16.4%, 95% CI: 6.6–26.1%). The results presented in Table 4 show that tackling, being-tackled and collisions were the most common activities associated with match injuries sustained by backs, whereas being-tackled, tackling and rucks were the most common for forwards. Players tackling (42.9%) was the match activity most often associated with concussion injuries; whereas, for knee-ligament (64.3%) and ankle-ligament (35.7%) injuries, players being tackled was the activity most often associated with injury.

**Table 4:** Match activity associated with injuries sustained by backs and forwards.

Match activity	% (95% confidence interval)		
	Backs	Forwards	ALL players
Collision	22.9 (8.9–36.8)	3.8 (0–9.1)	11.5 (4.8–18.2)
Kicking	0.0 (-)	0.0 (-)	0.0 (-)
Lineout	0.0 (-)	3.8 (0–9.1)	2.3 (0–5.4)
Maul	0.0 (-)	1.9 (0–5.7)	1.1)0–3.4)
Ruck	2.9 (0–8.4)	15.4 (5.6–25.2)	10.3 (3.9–16.7)
Running	5.7 (0–13.4)	9.6 (1.6–17.6)	8.0 (2.3–13.8)
Scrum	0.0 (-)	7.7 (0.4–14.9)	4.6 (0.2–9.0)
Tackled	25.7 (11.2–40.2)	32.7 (19.9–45.4)	29.9 (20.3–39.5)
Tackling	31.4 (16.0–46.8)	15.4 (5.6–25.2)	21.8 (13.2–30.5)
Other	11.4 (0.9–22.0)	9.6 (1.6–17.6)	10.3 (3.9–16.7)

## Discussion

As with all forms of rugby (27), forwards in the WXV competitions were significantly heavier and taller than backs. Players' body mass and stature showed increasing trends as a function of the WXV tier (WXV1 > WXV2 > WXV3). This situation is similar to that reported previously for the men's RWC, where players in the higher performing teams had higher body mass and stature (28). Players in the WXV competitions were significantly (*p* < 0.001) heavier (77.0 Kg; Table 1) than players appearing in the 2010 WRWC (73.7Kg) (18). This indicates that elite female players' body mass has increased over the past 15 years, which mirrors the situation that occurred during the professionalisation of men's rugby (28–31).

The overall incidence of injury recorded during the WXV competitions (49.5 injuries/1000 player-hours; Table 2) is significantly (*p* < 0.001) lower than that reported for international male players at the 2015 RWC (90.1 injuries/1,000 player-hours) and 2019 RWC (79.4 injuries/1,000 player-hours) competitions (13, 15). The incidence of injury in the WXV competitions is, however, similar to values reported (14) for men's international U20 players over the period 2008 to 2016 (average: 49.7 injuries/1,000 player-hours). Although the incidence of injury recorded during the WXV competitions was higher than that reported previously (18) for the 2010 WRWC (35.5 injuries/1,000 player-hours), the difference did not reach statistical significance (*p* = 0.078). The higher injury incidence recorded during the WXV competitions, compared to the 2010 WRWC, may be related to the WXV players' higher body mass, as greater body mass has been reported to lead to higher player momentum and greater impact forces during rugby contact events (31). At this time, the development and professionalisation of women's rugby has not yet experienced the significant increase in injury incidence observed previously during the professionalisation of men's rugby (32).

Players taking part in the WXV competitions were twice as likely to be injured in the second half of games compared to the first half. Similar differences were reported for the 2010 WRWC (18) (first half: 42.1%; second half: 57.9%; *p* = 0.168) and the men's 2015 RWC (13) (first half: 47.1%; second half: 52.9%; *p* = 0.276) and 2019 RWC (15) (first half: 41.7%; second half: 58.3%; *p* < 0.001). A statistically significant (*p* < 0.001) difference was also reported in men's international rugby-7s (33) (first half: 40.0%; second half: 60.0%; *p* < 0.001). In this case, the difference was ascribed to player fatigue (33), which is the most likely reason for the same situation observed in the other competitions. Fatigue is thought to increase match injury risk due to its detrimental effect on players' concentration, decision-making ability and reduction in proprioception and neuromuscular control during games (34, 35).

The high mean (42.2 days) and median (16.0 days) injury severities (Table 2) recorded during the WXV competitions were a consequence of the high proportion (9.9%) of major injuries (>90 days-absence) sustained. Despite this, WXV injury severities were similar to those reported previously for the 2010 WRWC (18) (mean: 55.0 days; median: 9.0 days). The mean and median severity values sustained during the WXV competitions are higher than the values reported for the men's 2015 RWC (mean: 29.8 days; median: 8.0 days) (13) and 2019 RWC (mean: 28.9 days; median: 9.0 days) (14). Although the high injury incidence and mean severity values during the WXV competitions result in a high injury burden value (2,089 injuries/1000 player-match-hours; Table 2), the value is similar to those observed previously during the 2010 WRWC (18) (1,953 days-absence/1,000 player-match-hours; *p* = 0.719) and men's 2015 and 2019 RWC (13, 15) (2,490 days-absence/1,000 player-match-hours; *p* = 0.352).

The three most common injury sub-locations reported during the WXV competitions (Table 3), the knee (22.8%), head/face (22.8%) and ankle (12.9%), accounted for 58.5% of all injuries sustained. These locations are the same as those reported for the 2010 WRWC (18) (knee: 28.2%, head/face: 23.1%, ankle: 12.8%), which accounted for 64.1% of all injuries sustained. The three most common injury sub-types sustained during the WXV competitions (Table 3: ligament-sprain: 35.6%, concussion: 14.9%, muscle-rupture: 9.9%), accounted for 60.4% of all injuries sustained. These injury types are different from those reported (18) for the 2010 WRWC (ligament-sprain: 28.2%, haematoma: 15.4%, meniscus lesion: 12.8%), which accounted for 56.4% of all injuries sustained. The most common injury sub-locations and sub-types at the women's WXV competitions are different from those reported for the men's 2015 and 2019 RWCs (13, 15). The most common injury locations reported at these competitions being the head/face (2015 RWC: 22.0%, 2019 RWC: 22.4%), knee (2015 RWC: 16.2%, 2019 RWC: 11.9%) and posterior thigh (2015 RWC: 10.4%; 2019 RWC: 12.6%). The most common injury types sustained at the men's 2015 and 2019 RWC were ligament-sprain (2015 RWC: 23.1%; 2019 RWC: 21.7%), muscle-rupture (2015 RWC: 23.1%; 2019 RWC: 20.3%) and concussion (2015 RWC: 13.9%; 2019 RWC: 15.4%).

In addition to the short-term, time-loss consequences of injury, there are potential long-term consequences associated with some injuries. Links between knee-ligament injuries sustained in sport and post-career osteoarthritis have been reviewed by the UK Industrial Injuries Advisory Council (36). Although this report focused on retired male and female footballers, it provided general information and conclusions about post-career, sport-related osteoarthritis, which is equally relevant to retired rugby players. For example, 82% of female footballers who had sustained an anterior cruciate ligament injury were reported to exhibit radiographic osteoarthritis 12 years post-injury compared to 37% in a control sample (36). Adverse brain health among retired rugby players is an active area of research but there is little information available, at present, for retired female players. A recent study (37) of 200 mid-life ex-rugby players (male:90%; female: 10%) with brain health concerns reported high rates for subjective cognitive complaints but there was little evidence of differences in objective cognitive impairments and no evidence of dementia among the players. Reporting when injured players are removed from match play has been a fundamental aspect of all World Rugby epidemiological studies since the 2007 RWC (11). Two-thirds of the confirmed concussions sustained by players during the WXV competitions were identified during the game and the players were removed from play immediately; this is higher than that previously reported (18) for the women's 2010 RWC (38.5%) but is similar to values reported (13, 15) for the men's 2015 RWC (75.0%) and 2019 RWC (77.3%). The complexities associated with earlier identification of delayed-onset concussions have been discussed previously (38) and these issues remain unresolved.

The tackle is the match activity that has most often been associated with injury (39, 40) and, in particular, with concussion; this was also the case in the present study. World Rugby has explored the potential benefits of lowering the legal tackle height to the line of the armpit and to below the base of the sternum in community rugby, in order to reduce the incidence of concussion (41, 42). Although the incidences of head-to-head and shoulder-to-head impacts were reduced in men's amateur rugby, no significant change occurred in the incidence of concussion (41). More recently, a study in women's amateur rugby reported similar reductions in head-to-head and shoulder-to-head impacts but again there was no significant change in concussion rate (42).

The overall risk of injury in rugby is defined by the incidence, severity and nature of injuries sustained by players and by the match and training load that players are exposed to (43). To address the second of these risk factors, World Rugby has proposed, with effect from 2026, annual exposure limits (44) for match and training activities undertaken by elite male and female rugby players (45). Prior to the present study, there was little epidemiological information available covering women's international rugby-15s. The results reported here support the need to collect and evaluate longitudinal epidemiological data separately for female rugby players. Strengths of the present study are that the data were collected according to the international consensus statement for injury surveillance studies in rugby and all data were collected and reported by team medical staff. The results presented, therefore, provide a valid source of information about the risk of injury at the international level of women's rugby-15s and they provide a base line from which to assess the impact of World Rugby's proposal for annual exposure limits in women's elite rugby.

## Data Availability

The detailed datasets used in the preparation of this article are not available due to the confidential nature of players' personal medical data. Requests for additional information should be directed to CF.
